# Synaptic Components, Function and Modulation Characterized by GCaMP6f Ca^2+^ Imaging in Mouse Cholinergic Myenteric Ganglion Neurons

**DOI:** 10.3389/fphys.2021.652714

**Published:** 2021-08-02

**Authors:** Joseph F. Margiotta, Kristen M. Smith-Edwards, Andrea Nestor-Kalinoski, Brian M. Davis, Kathryn M. Albers, Marthe J. Howard

**Affiliations:** ^1^Department of Neurosciences, College of Medicine and Life Sciences, University of Toledo, Toledo, OH, United States; ^2^Department of Neurobiology, University of Pittsburgh School of Medicine, Pittsburgh, PA, United States; ^3^Department of Surgery, College of Medicine and Life Sciences, University of Toledo, Toledo, OH, United States

**Keywords:** mouse, myenteric, synapse, nicotinic acetylcholine receptor (nAChR), ionotropic serotonin receptor (5-HT3R)

## Abstract

The peristaltic contraction and relaxation of intestinal circular and longitudinal smooth muscles is controlled by synaptic circuit elements that impinge upon phenotypically diverse neurons in the myenteric plexus. While electrophysiological studies provide useful information concerning the properties of such synaptic circuits, they typically involve tissue disruption and do not correlate circuit activity with biochemically defined neuronal phenotypes. To overcome these limitations, mice were engineered to express the sensitive, fast Ca^2+^ indicator GCaMP6f selectively in neurons that express the acetylcholine (ACh) biosynthetic enzyme choline acetyltransfarse (ChAT) thereby allowing rapid activity-driven changes in Ca^2+^ fluorescence to be observed without disrupting intrinsic connections, solely in cholinergic myenteric ganglion (MG) neurons. Experiments with selective receptor agonists and antagonists reveal that most mouse colonic cholinergic (i.e., GCaMP6f^+^/ChAT^+^) MG neurons express nicotinic ACh receptors (nAChRs), particularly the ganglionic subtype containing α3 and β4 subunits, and most express ionotropic serotonin receptors (5-HT_3_Rs). Cholinergic MG neurons also display small, spontaneous Ca^2+^ transients occurring at ≈ 0.2 Hz. Experiments with inhibitors of Na^+^ channel dependent impulses, presynaptic Ca^2+^ channels and postsynaptic receptor function reveal that the Ca^2+^ transients arise from impulse-driven presynaptic activity and subsequent activation of postsynaptic nAChRs or 5-HT_3_Rs. Electrical stimulation of axonal connectives to MG evoked Ca^2+^ responses in the neurons that similarly depended on nAChRs or/and 5-HT_3_Rs. Responses to single connective shocks had peak amplitudes and rise and decay times that were indistinguishable from the spontaneous Ca^2+^ transients and the largest fraction had brief synaptic delays consistent with activation by monosynaptic inputs. These results indicate that the spontaneous Ca^2+^ transients and stimulus evoked Ca^2+^ responses in MG neurons originate in circuits involving fast chemical synaptic transmission mediated by nAChRs or/and 5-HT_3_Rs. Experiments with an α7-nAChR agonist and antagonist, and with pituitary adenylate cyclase activating polypeptide (PACAP) reveal that the same synaptic circuits display extensive capacity for presynaptic modulation. Our use of non-invasive GCaMP6f/ChAT Ca^2+^ imaging in colon segments with intrinsic connections preserved, reveals an abundance of direct and modulatory synaptic influences on cholinergic MG neurons.

## Introduction

The enteric nervous system (ENS) generates circuit activity that produces peristalsis *via* rhythmic contraction and relaxation of intestinal circular and longitudinal smooth muscles (Costa et al., 2000). To do so, relevant synaptic circuits in the myenteric plexus (MP) integrate and modulate activity from inputs both outside the ENS and from intrinsic sensory neurons and interneurons to control the output of circular and longitudinal myenteric motor neurons (Gwynne and Bornstein, 2007; Furness et al., 2014). Myenteric ganglion (MG) neurons display an array of structural, functional, and biochemical phenotypes with the latter biochemical code exemplified by expression of diverse neurotransmitters. Acetylcholine (ACh) is the major neurotransmitter in MG and intrinsic motor and primary sensory neurons, as well as intrinsic ascending interneurons and some descending interneurons all synthesize ACh, utilizing the cholinergic enzyme choline acetyltransferase (ChAT) (Galligan et al., 2000; Lomax and Furness, 2000; Furness, 2012). Moreover, electrophysiological studies using guinea pig or mouse intestinal *ex vivo* preparations have shown that fast cholinergic synaptic signaling involving ACh release from presynaptic neuron terminals to activate ionotropic (i.e., nicotinic) ACh receptors (nAChRs) on postsynaptic neurons is a primary means of neurotransmission in MG (Galligan and North, 2004; Gwynne and Bornstein, 2007; reviewed by Foong et al., 2015). Such studies provide detailed information about the function of MG synapses and their component receptors, but prevailing electrophysiological approaches involve stripping away circular and mucosal plexus layers to attain electrode access, thereby disrupting intrinsic connections and limiting conclusions to the most accessible neurons receiving the most localized inputs.

Cell imaging based on fluorescent membrane-permeable or genetically-encoded Ca^2+^ indicators (GECIs) presents an alternative for assessing synaptic transmission in whole tissues that has notable advantages over electrophysiology. The unstripped, intact intestinal segments used for imaging preserve intrinsic synaptic connections and are sufficiently thin to allow optical detection of evoked and spontaneous neuronal Ca^2+^ signals. Moreover, available mice strains make it possible to co-express GECIs with phenotypic enzymes, allowing GECI distribution and subsequent interrogation confined to neurons having a specific neurotransmitter phenotype. Disadvantages are that intracellular Ca^2+^ indicators provide an indirect assay of transmembrane potential or current because they reflect both Ca^2+^ influx and release from intracellular stores. On and off rates of Ca^2+^ binding also result in much slower temporal resolution than provided by electrophysiology. These disadvantages are mitigated, albeit not entirely, by the advent of sensitive, fast-kinetics GECIs typified by GCaMP6f (Chen et al., 2013). Notably, in studies of hippocampal and visual cortex layer 2/3 pyramidal neurons that combined electrophysiological and imaging approaches, transient GCaMP6f Ca^2+^ signals could be correlated with membrane potential changes, and did so with temporal resolution sufficient to align 1:1 with single local field potentials and action potentials, respectively (Chen et al., 2013; Li et al., 2019a). In accord with its utility, GCaMP Ca^2+^ imaging has been employed previously to assess the role of nAChRs in stimulus evoked synaptic transmission in the mouse colon (Foong et al., 2015; Li et al., 2019b). Moreover, and contrasting with electrophysiological results from MG neurons in stripped intestinal preparations (Stebbing and Bornstein, 1996; Fung et al., 2018; but see Nurgali et al., 2004) GCaMP imaging reveals spontaneous Ca^2+^ transients attributable to nAChR-mediated synaptic activity in MG neurons in unstripped colon preparations (Hennig et al., 2015; Smith-Edwards et al., 2019, 2020). Only Hennig et al. (2015) focused on cholinergic (ChAT^+^) neurons, however, and a slower GECI (GCaMP3) was employed, obviating a full analysis of the Ca^2+^ transients’ receptor dependence and their links to endogenous synaptic transmission.

Here, we used GCaMP6f to characterize synaptic receptors as well as spontaneous and evoked synaptic activity in colonic cholinergic MG neurons by engineering mice to confine its expression to cholinergic (ChAT^+^) neurons. Our results reveal that 69% of cholinergic (i.e., ChAT^+^/GCaMP6f^+^) MG neurons from intact colon segments possess functional nAChRs particularly the ganglionic subtype containing α3 and β4 subunits, and a similar fraction possess functional ionotropic serotonin type 3 receptors (5-HT_3_Rs). The neurons also display spontaneous and nerve-stimulated Ca^2+^ transients indicative of considerable intrinsic and readily evoked synaptic activity, mediated in both cases by nAChRs or/and 5-HT_3_Rs. In addition, such activity can be modulated by altering neurotransmitter release with α7-nAChR or pituitary adenylate cyclase-activating polypeptide (PACAP) receptor agonists. Our results, using mouse colon segments with most intrinsic connections preserved, reveal abundant and diverse synaptic interactions affecting cholinergic MG neurons. While confined to the ENS, the results further indicate that GECIs can be used in semi-intact preparations to probe the function and modulation of synapses on genetically targeted neurons throughout the nervous system.

## Materials and Methods

### Generation of Mice Expressing Genetically-Encoded Ca^2+^ Indicators

Male and female mice aged 3–6 months were studied. For nearly all experiments, mouse strains were cross-bred to express GCaMP6f, selectively in cholinergic enteric neurons. To do so mice that contain a floxed-STOP GCaMP6f construct within the ROSA26 locus (Ai95, RRID:Addgene_61579; RRID:IMSR_JAX:028865) were crossed with mice that express Cre recombinase under control of the choline acetyltransferase (ChAT) promoter (ChAT-IRES-Cre, RRID:IMSR_JAX:006410). For some experiments involving electrical stimulation of MG connectives and nAChR/5-HT_3_R co-expression, mice cross-bred to express GCaMP6s in cells expressing E2a, an upstream activator of neural lineage genes, were used. To do so, mice containing a floxed-STOP-GCaMP6s sequence in the Rosa26 locus (Ai96 mice, RRID:IMSR_JAX:028866) were crossed with E2a+/E2a-Cre mice (RRID:IMSR_JAX:003724).

### Imaging Ca^2+^ Signals From Cholinergic Myenteric Neurons in Mouse Proximal Colon

Excised proximal colon segments were opened along the mesenteric border and pinned mucosal side down onto a Sylgard surface (Dow Chemical) lining a glass coverslip attached to the bottom of a plastic imaging chamber containing Krebs solution (in mM: 118 NaCl, 4.7 KCl, 1.2 MgSO4, 2.5 CaCl_2_, 1.2 mM KH_2_PO_4_, 25 NaHCO_3_, and 11 glucose). The explant segments were examined at 20–22°C in Krebs solution alone (Control) or in Krebs solution containing test reagents (e.g., antagonists; Test). The solutions were kept in separate refillable, height-adjustable 60 ml syringes and bubbled with Carbogen (95% O_2_/5% CO_2_) to oxygenate and achieve pH 7.4. A control or test solution was selected by opening a stopcock valve attached to the relevant syringe and the solution gravity-fed *via* Silastic tubing (1.02 mm ID, Dow Corning) to a four-input, one-output port manifold (MP-4 Series, Warner Instruments) and thence to the chamber at 1–2 ml/min. GCaMP positive neurons were detected by their basal Ca^2+^ fluorescence using an Olympus BX-51 microscope fitted with an Olympus UMPlanFL 20×/0.5 NA water-immersion objective and epifluorescence optics consisting of a 470 nm 3W LED source (Mightex Systems, BLS-LCS-0470-03-22) control module (Mightex Systems, BIOLed BLS-IO04-US) and an appropriate 470–490 nm excitation and 520 nm emission filter cube set (Olympus, U-MNB2). E2a^+^/GCaMP6s^+^ myenteric neurons were distinguished from other cell types based on the following characteristics: (1) spatial location within myenteric ganglia, (2) cell body shape and size, and (3) the latency and upstroke rate of response to electrical stimulation (Boesmans et al., 2013; Smith-Edwards et al., 2019, 2020).

Evoked and spontaneous changes in the intensity of GCaMP-mediated Ca^2+^ fluorescence were acquired in 12-bit images using a 1.44 Megapixel CMOS camera capable of capturing at up to 80 frames/s (Prime 95B, Teledyne Photometrics) controlled by MetaMorph software (Version 7.10.1.161, Molecular Devices). Image stacks of 400–1800 frames were processed, and when necessary motion-corrected, using Fiji software (Version 2.0.0-rc-65/1.52a) (Schindelin et al., 2012). The stacks were segmented by drawing regions of interest (ROIs) around an area of representative background fluorescence (F_0_), and around MG neuron somas that (1) displayed basal levels of GCaMP-mediated Ca^2+^ fluorescence intensity (F) and (2) displayed detectable F elevation in response to applied agonist or that displayed spontaneous, transient increases in F. The mean F values of ROIs in each frame were acquired and exported to Excel (Microsoft, 2016) spreadsheets where the time-dependent, background-subtracted and normalized changes in specific neuronal Ca^2+^ fluorescence [(F − F_0_)/F_0_ = ΔF/F_0_] were calculated for each neuron per frame (typically 15 neurons in 1800 frames at 40 Hz) and used for subsequent analyses. The net amplitudes of peak ΔF/F_0_ increases induced by agonists or evoked by electrical stimulation (A_A,Peak_ or A_E,Peak_) were obtained from the records by subtracting basal from peak ΔF/F_0_ values (A_A,Peak_ or A_E,Peak_ = Peak ΔF/F_0_ − Basal ΔF/F_0_).

The functional repertoire of accessible ionotropic neurotransmitter receptors on cholinergic MG neurons was assessed from A_A,Peak_ responses evoked by fast application of ionotropic receptor agonists and their inhibition by incubation with selective antagonists. Agonists were dissolved in Krebs solution and focally applied by pressure microperfusion (5 psi, 10 s; *via* Picospritzer II; Parker Instrumentation Corp.) from blunt glass micropipettes (5–10 μm diameter) positioned within 50 μm of an adjacent MG. Dimethylphenyl-piperazinium (DMPP, 10 μM) was used to assay functional nicotinic acetylcholine receptors (nAChRs) because it potently activates nAChRs on MG neurons (Zhou et al., 2002). Functional ionotropic serotonin (5-HT) responses were evoked by applying 5-hydroxytryptamine (5-HT, 10 μM) in the same fashion. To verify receptor specificity, test Krebs solutions containing nAChR or 5-HT_3_R selective antagonists were applied by the perfusion system to colon segments for pre-incubation times indicated in the text, followed by re-testing with agonist as above. The nAChR channel blocking antagonist hexamethonium (Hex, 100 μM) and the competitive antagonist D-tubocurarine (dTC, 100 μM) were used singly or in combination to inhibit nAChR function, and SR16584 (40 mM) used to selectively inhibit function of the ganglionic nAChR subtype, containing α3 and β4 subunits. The dependence of 5-HT responses on ionotropic receptors was validated using Ondansetron (10–20 μM) an antagonist specific for 5-HT_3_Rs (Machu, 2011).

The frequency and amplitude of spontaneous Ca^2+^ transients (F_S_ and A_S,Peak_) were obtained from the Microsoft Excel ΔF/F_0_ data values using a PeakCaller script (Artimovich et al., 2017) run under Matlab (R2018b). Because antagonists reduced Ca^2+^ transient frequency to low levels, PeakCaller scored the residual tiny spikes as valid events. Thus, all PeakCaller event lists were filtered in Excel to include only criterion events having A_S,Peak_ amplitudes >0.015, and decay half times >100 ms. F_S_ values (events/s; Hz) were obtained by dividing the number of the resulting criterion-level events for each neuron by the record length (usually 30–45 s). The kinetics of Ca^2+^ transients were determined from selected temporally isolated transients displayed graphically in Excel. To do so, the time and amplitude of a Ca^2+^ transient (A_S,Peak_ = Peak ΔF/F_0 –_ Basal ΔF/F_0_) were first identified. The event rise half-time (T_R,1/2_) was then calculated from the net time required for ΔF/F_0_ to rise from 2 to 4 SD above baseline to 50% of A_S,Peak_ and then the decay-half time (T_D,1/2_) calculated from the net time required to fall from A_S,Peak_ by 50%. Standard pharmacological tests were conducted to determine the activity and synaptic dependence of the Ca^2+^ transients. To determine whether the Ca^2+^ transients required Na^+^ dependent action potentials, tetrodotoxin (TTX, 1 μM) and/or lidocaine (Lido, 2 mM) were applied to block voltage gated Na^+^ channels. To determine whether the Ca^2+^ transients arose from synaptic activity, pre- and postsynaptic function was perturbed. Presynaptic function was inhibited using ω-conotoxin-MVIIC (ω-CTx) a selective blocker of P/Q- and N-type Ca^2+^ channels (Ca_V_2.1 and Ca_V_2.2, respectively) prevalent on presynaptic terminals (Wu and Saggau, 1995; McDonough et al., 1996) and responsible for initiating neurotransmitter release (Mochida, 2018). Postsynaptic function was perturbed using selective antagonists of ionotropic AChRs (Hex and dTC) and 5-HT_3_ neurotransmitter receptors (Ondansetron or LY278584) as described above.

To probe for synaptic inputs onto cholinergic MG neurons, single current pulses (1500 μA, 100–200 μs) or pulse trains (20 at 10 Hz) were delivered to MP inter-ganglionic connectives from a digital constant-current stimulator (Multichannel Systems STG 4000) and applied *via* a focal concentric bipolar electrode (550 μm OD, CBJRJ75, FHC Inc.) positioned 5–6 mm oral to the imaging site, and the evoked Ca^2+^ responses acquired at 80 Hz. As described above for spontaneous Ca^2+^ transients, the responses evoked by connective stimulation were evaluated for net peak amplitude (A_E,Peak_) and, in cases of responses evoked by single pulses, for rise and decay half-times (T_R,1/2_ and T_D,1/2_). The synaptic dependence of the stimulus evoked responses was tested using selective antagonists for presynaptic Ca^2+^ channels or postsynaptic ionotropic neurotransmitter receptors as described above. Single stimulus trials were used to infer whether such evoked synaptic responses reflected activation of mono- versus polysynaptic pathways, and based on calculating overall response latency determined from the time elapsed between stimulus application and onset of initial response, with initial response defined as 2–4× SD above pre-stimulus ΔF/F_0_. Estimates of synaptic transmission delay times were obtained by subtracting the conduction time required for impulses to travel from stimulation to recording site from the overall response latency, with conduction time determined from the measured distance between stimulating electrode and imaging site (5–6 mm) and the previously determined conduction velocity (0.55 mm/ms) of a longitudinally projecting axon action potential (Stebbing and Bornstein, 1996).

#### Statistics and Drug Testing

The effects of test drugs on response parameters (i.e., A_E,Peak_, A_S,Peak_, F_S_, T_R,1/2_, and T_D,1/2_) were assessed by comparing values obtained from cells incubated with the drug (Test) relative to those *obtained from the same cells* prior to drug treatment (Control). Except where noted, *N* ≥ 2 explants were assessed for each drug treatment. The values obtained for control and test conditions were considered significantly different if *p* < 0.05, with *p* determined using Student’s paired two-tailed *t*-test (Prism 4; GraphPad Software, San Diego, CA, United States). Test drugs (with their abbreviations, concentrations, minimum pre-incubation times, targets, and actions thereupon) are listed in Table 1.

**TABLE 1:** Test drugs were applied to colon explants in Krebs solution either by the perfusion system^1^ or by pipetting them directly into the chamber^2^.

Drug	Abbreviation	Final concentration in Krebs	Incubation time (min)	Target and drug action
D-tubocurarine^1^	dTC	100 μM	10–15	nAChR antagonist
Hexamethonium^1^	Hex	100 μM	10–15	nAChR antagonist
SR16584^1^	SR	40 μM	10–15	α3β4-nAChR antagonist
Ondansetron^1^	Ondansetron	20 μM	10–15	5-HT_3_R antagonist
LY278584^1^	LY278584	10 μM	20	5-HT_3_R antagonist
Methyllycaconitine^1^	MLA	50 nM	60	α7-nAChR antagonist
Tetrodotoxin^1^	TTX	1 μM	15	Na^+^ channel blocker
Lidocaine^1^	Lido	2 mM	15	Na^+^ channel inhibitor
ω-conotoxin-MVIIC^2^	ω-CTx	100–200 nM	60	P/Q and N-type Ca^2+^ channel blocker
Pituitary adenylate cyclase activating polypeptide^2^	PACAP	100 nM	5	PAC_1_R, VPACR agonist

### RNA Detection and Immunostaining Methods

mRNAs encoding nAChR subunits were detected with an RNAscope in-situ hybridization assay (ACD Biotechne) using the manufacturer’s ready-made probes for mammalian α7- and α3-nAChR subunits (465161-C2 and 449191, respectively) applied to fresh-frozen colon tissue sections (14 μm) with appropriate controls for specificity employed. The method provided by the manufacturer was followed for fresh frozen fluorescent visualization of tagged probes. All sections were co-labeled with DAPI to visualize cell nuclei and overall tissue morphology. Images were acquired on a Leica TCS SP5 laser scanning confocal microscope at 20× magnification as described below.

Immunostaining was performed on colon explant whole-mounts as reported previously (Hendershot et al., 2008; Lei and Howard, 2011). Briefly, intact colons were flushed, and fixed in 4% paraformaldehyde freshly prepared in Dulbecco’s phosphate buffered saline (DPBS) for 1–2 h at RT or overnight at 4°C. Following extensive washing in DPBS, tissue samples were incubated in a buffer containing 0.1M Tris, 1.5% NaCl, 0.5% TX-100 (TNTX, 3 × 30 min) blocked in TNTX containing 20% horse serum (TNTXHS, 2 × 1 h with shaking) followed by rinsing in TNTX. Primary antibodies were applied in TNTXHS for 48 h at 4°C with rocking. Neurons expressing 5-HT were identified by co-labeling with neuron-specific HuC/D antibody (ANNA-1; 1:20,000. Gift from Dr. VA Lennon, The Mayo Clinic) and 5-HT specific antibody (Immunostar, 20080, 1:1000). Following washing in TNTXHS (3 × 1 h), and washing in TNTX (3 × 10 min) secondary antibody was applied in TNTXHS for 48 h at 4°C with rocking. Following washing in TNTX (6 × 10 min) tissues were mounted in Fluoromount-G (Thermo Fisher Scientific, Waltham, MA, United States) and visualized using a Leica TCS SP5 laser scanning confocal microscope (Leica Microsystems, Bannockburn, IL, United States) equipped with continuous-wave solid state lasers (458, 488, 514, 561, and 633 nm) and a titanium-sapphire tunable (705–980 nm) multiphoton laser (Coherent, Santa Clara, CA, United States). Images were acquired at 512 × 512 resolution in the XYZ planes as a single field of view or as tile scans (XYZS) in 1 μm steps with 20× (NA 0.70), 40× (NA 1.25), or 63× (NA 1.4) objectives using an automated scanning stage. To minimize spectral overlap in the emission spectrum, images were acquired with the LAS AF software in sequential scan mode.

## Results

### Cholinergic MG Neurons Express Functional nAChRs and 5-HT_3_Rs

**FIGURE 1:**
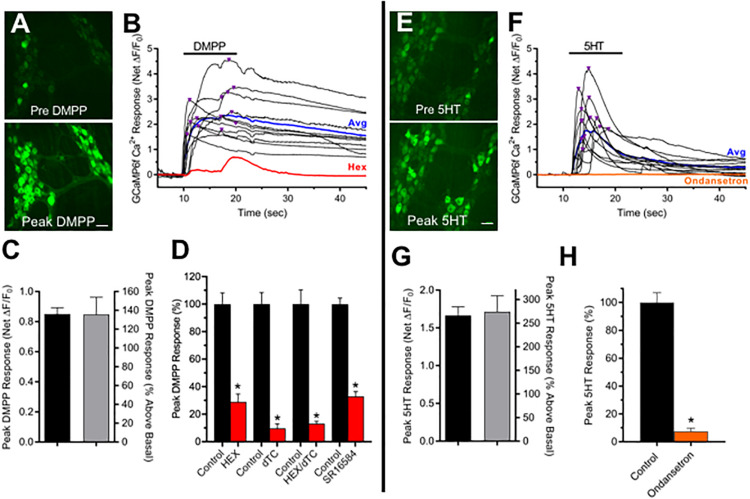
Most cholinergic MG neurons express functional nAChRs and 5-HT_3_Rs. **(A–D)** nAChRs. **(A)** Panels depict neuronal GCaMP6f Ca^2+^ fluorescence increases from basal levels (top panel) to peak levels (bottom panel) associated with DMPP application. Panels depict two MG, each containing several GCaMP6f^+^/ChAT^+^ neurons. Scale bar at bottom represents 40 μm. **(B)** Time course of Ca^2+^ fluorescence changes in 13 of the neuron somas depicted in **A** black traces) in response to focally applied DMPP (black bar; 20 μM, 20 psi). Inverted triangles above the traces depict the timing of net peak ΔF/F_0_ responses (A_A,Peak_). The response average from these neurons and that of the same neurons after incubation with hexamethonium (Hex, 100 μM) are depicted by the blue and red traces, respectively. **(C)** Global summary of DMPP responses. A total of 69 ± 6% of cholinergic MG neurons tested from *N* = 15 explants responded to 10 μM DMPP, doing so with an average net peak ΔF/F_0_ amplitude (A_A,Peak_) of 0.85 ± 0.04 (*n* = 321) representing a 135 ± 18% increase above basal ΔF/F_0_. **(D)** Pharmacology of DMPP responses. Incubation with generic nAChR antagonists Hex (100 μM), dTC (100 μM) or their combination (Hex/dTC), or with SR16584 (40 μM) an antagonist selective for α3, β4-containing nAChRs for 10–15 min reduced A_A,Peak_ doing so, respectively, to 29 ± 6% (*n* = 15), 10 ± 3% (*n* = 15), or 13 ± 2% (*n* = 29), or 33 ± 4% (*n* = 75) of levels obtained from the same neurons before antagonist application (black bars, Control). **(E–H)**. 5-HT_3_Rs. The fields in **(E)** each depict two myenteric ganglia containing several GCaMP6f^+^/ChAT^+^ neurons. Neuronal Ca^2+^ fluorescence increases from basal levels (top) to high levels following exposure to 10 μM 5-HT (bottom). Scale bar at bottom represents 40 μm. **(F)** Time course of GCaMP6f Ca^2+^ fluorescence responses to focally applied 5-HT (black bar, 10 μM, 20 psi) in 13 of the neurons depicted in **(A)** (black traces). Inverted triangles above the traces depict the timing of A_A,Peak_. The response average from these neurons and that of the same neurons after incubation with Ondansetron (20 μM; 10–15 min) are depicted by the blue and orange traces, respectively. **(G)** Global summary of 5-HT responses. A total of 62 ± 6% of cholinergic MG neurons tested from *N* = 3 explants responded to 5-HT, doing so with an average A_A,Peak_ of 1.66 ± 0.12 (*n* = 60) representing a 274 ± 34% increase above basal ΔF/F_0_. **(H)** Pharmacology of 5-HT responses. Incubation with the 5-HT_3_R-selective antagonist Ondansetron (20 μM; 10–15 min) reduced A_E,Peak_ (orange bar) doing so to 8 ± 2% (*n* = 45) of levels obtained from the same neurons before antagonist application (black bar, Control). Asterisks in this and subsequent figures indicate a significant difference between values for *n* neuron pairs obtained *after* versus *before* drug treatment (*p* < 0.05, Student’s paired *t*-test).

Ganglionic nAChRs are crucial components of autonomic synapses. To probe for functional nAChRs, cholinergic ChAT^+^/GCaMP6f^+^ MG neurons, identified by basal fluorescence indicative of resting Ca^2+^ levels, were challenged with DMPP. Focal pressure application of DMPP increased neuronal Ca^2+^ fluorescence (ΔF/F_0_) in all ChAT^+^/GCaMP6f^+^ colon explants (*N* = 15) doing so in 69% of cholinergic MG neurons within the field of view (Figures 1A–D). The DMPP responses detected by GCaMP6f featured rapid increases in neuronal Ca^2+^ fluorescence, reaching net agonist-induced peak amplitude A_A,Peak_ = 0.85, representing a 135% increase above basal ΔF/F_0_, usually within 5 s. Treatment with nAChR antagonists inhibited subsequent DMPP responses. The nAChR channel blocker hexamethonium (Hex, 100 μM), the competitive nAChR antagonist D-tubocurarine (dTC, 100 μM) or a combination of the two (Hex/dTC) each inhibited the DMPP response in 100% of neurons tested, causing 71, 90, or 87% respective reductions in DMPP-evoked A_A,Peak_ when compared to paired control neurons tested prior to antagonist treatment. Prominent ganglionic nAChR subtypes on MG and other autonomic neurons contain α3 and β4 subunits (Mandelzys et al., 1994; Nai et al., 2003; Galligan and North, 2004; Gwynne and Bornstein, 2007; Foong et al., 2015). Such receptors are termed α3β4^∗^-nAChRs (Lukas et al., 1999) with the asterisk indicating they may also contain α5 and β2 subunits (Vernallis et al., 1993; Mandelzys et al., 1994; Nai et al., 2003). Treatment with SR16584 (SR, 40 μM) an antagonist selective for nAChRs containing α3 and β4 subunits (Zaveri et al., 2010) inhibited subsequent DMPP responses in 95% of neurons tested, reducing DMPP-evoked A_A,Peak_ by 67% overall. Taken together, these results indicate that nAChRs underlie the DMPP response of MG neurons, with the majority of the response mediated by α3β4^∗^-nAChRs. Because 5-HT_3_Rs like nAChRs, are Na^+^/Ca^2+^-permeable excitatory ionotropic receptors, and known to be present on MG neurons (Mawe et al., 1986; Zhou and Galligan, 1999; Galligan et al., 2000; Glatzle et al., 2002) we tested whether they could be detected with GCaMP6f Ca^2+^ imaging. Similar to the response incidence for DMPP, 62% of MG neurons from ChAT^+^/GCaMP6f^+^ colon explants responded to 5-HT (Figures 1E–H) in this case resulting in A_A,Peak_ responses of 1.66 that were 274% above basal ΔF/F_0_. The 5-HT induced responses reflect activation of 5-HT_3_Rs. Pre-incubation and testing in Ondansetron (20 μM) a potent 5-HT_3_R-selective antagonist (Machu, 2011) inhibited 5-HT induced responses in 100% of neurons tested, reducing A_A,Peak_ by 92%. These results demonstrate that most cholinergic MG neurons express nAChRs, with the majority of the response to DMPP represented by α3β4^∗^-nAChRs, and that most also express 5-HT_3_Rs, thereby indicating that a sizeable fraction of the neurons likely express both receptor types.

### Cholinergic MG Neurons Display Spontaneous Ca^2+^ Transients

**FIGURE 2:**
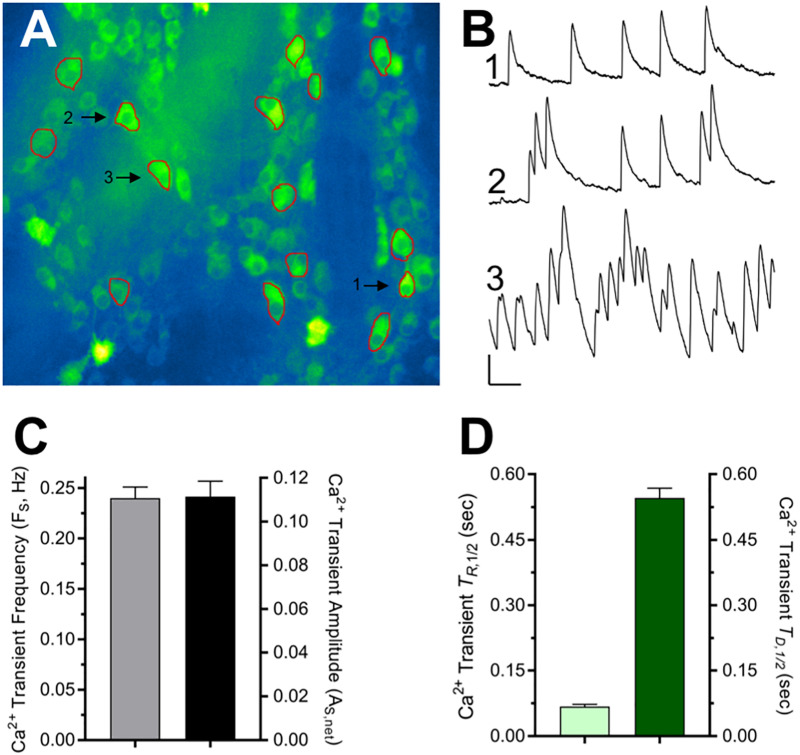
Properties of spontaneous Ca^2+^ transients displayed by cholinergic MG neurons. **(A)** Single frame image of ChAT^+^/GCaMP6f^+^ neurons within two MG (see accompanying time-lapse Video in Supplementary Figure 1.). ROIs drawn around 15 neuron somas chosen for analysis are indicated in red. **(B)** Traces show three representative types of Ca^2+^ transient activity from neurons labeled 1, 2, and 3 in **(A)** that displayed transients that were isolated (1) moderately clustered (2), or tight clustered (3). Calibration bars: vertical = 0.1 net ΔF/F_0_, horizontal = 5 s. **(C)** Overall characteristics of the spontaneous Ca^2+^ transients. Spontaneous Ca^2+^ transient frequency (F_S_) was 0.240 ± 0.011 Hz (*n* = 220 from *N* = 14 explants). Ca^2+^ transient net peak amplitude (A_S,Peak_ = Peak ΔF/F_0_ – Basal ΔF/F_0_) was 0.111 ± 0.007 evaluated using temporally isolated Ca^2+^ transients selected at random (*n* = 95; *N* = 7). **(D)** For the same neurons where A_S,Peak_ was determined, Ca^2+^ transient kinetics were assessed from the event rise and decay half-times (T_R,1/2_ = 67 ± 5 ms and T_D,1/2_ = 546 ± 23 ms) measured from traces displayed and analyzed in Excel (Microsoft, 2016) as described in section “Materials and Methods.”

Spontaneous fast-onset, slow decay Ca^2+^ transients have been detected previously in neurons and attributed to single action potentials and even subthreshold synaptic activity (Chen et al., 2013; Li et al., 2019a). Such Ca^2+^ transients were detected in virtually all intact ChAT^+^/GCaMP6f^+^ colon explants (96%, *N* = 23) and observed in approximately 25% of cholinergic MG neuron somas, occurring both in clusters and as temporally isolated events (Figure 2 and Supplementary Figure 1). On a per neuron basis, the spontaneous Ca^2+^ transients occurred at an average frequency (F_S_) of 0.24 Hz, and the mean net peak ΔF/F_0_ amplitude of temporally isolated Ca^2+^ transients (A_S,Peak_) was 0.11, a level 8× or 16× smaller than that of the global cellular responses to DMPP or 5-HT, respectively (Figure 1). Temporally isolated Ca^2+^ transients were amenable to kinetic analyses, displaying mean half-rise times (T_R,1/2_) of 67 ms and half-decay times (T_D,1/2_) of 546 ms. The observed T_R,1/2_ is consistent with previous values obtained for GCaMP6f-mediated Ca^2+^ transients induced by single action potentials in mammalian cortical neuron somas (45–100 ms; see Figure 1 and Supplementary Table 3 in Chen et al., 2013). The observed T_D,1/2_, however, is 2.7–3.8 times longer than that reported for single action potential induced Ca^2+^ transients in the same cortical neurons (142–200 ms). Because the higher buffering capacity and larger size of MG neurons can account for most of this discrepancy (see section “Discussion”) these results indicate that GCaMP6f is sufficiently sensitive to report changes in intracellular Ca^2+^ that result from signals on the order of single action potentials in cholinergic MG neurons.

**FIGURE 3:**
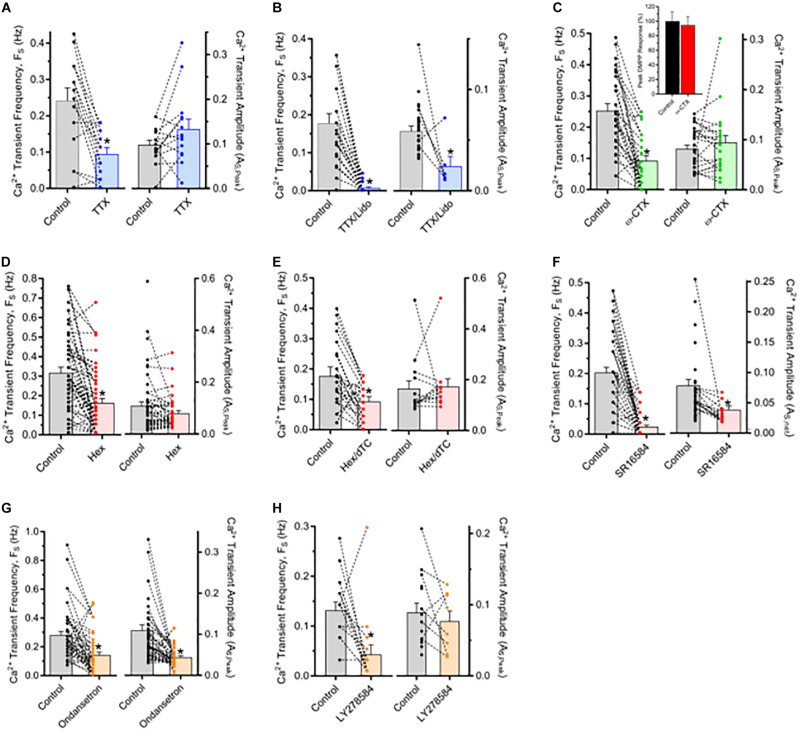
Spontaneous Ca^2+^ transients depend on impulse-driven synaptic transmission mediated by nAChRs and 5-HT_3_Rs. The results depict F_S_ and A_S,Peak_ measurements from neuron pairs in ChAT^+^/GCaMP6f^+^ MG explants tested before (Control) and after treatment with antagonists (as indicated). Bar graphs depict F_S_ (left) and A_S,Peak_ (right) averages while the superimposed dot plots and connecting lines depict their values from individual neurons before and after treatment. In the legend and text changes in F_S_ and A_S,Peak_ are presented as a *per cent* difference between values for test and control group neurons within a field. Note that A_S,Peak_ is represented for neurons from only those records where F_S_ > 0. **(A, B)** Ca^2+^ transients depend on impulses requiring voltage-gated Na^+^ channels. **(A)** Tetrodotoxin (TTX; 1 μM, 15 min) reduced F_S_ by 61 ± 7% (*n* = 15; *N* = 1) without affecting A_S,Peak_. **(B)** Combining TTX with 2 mM Lidocaine (TTX/Lido, 15 min) to also block TTX-resistant Na^+^ channels nearly abolished the Ca^2+^ transients resulting in a 96 ± 2% reduction in F_S_ (*n* = 27; *N* = 2) that was accompanied by a 60 ± 18% reduction in A_S,Peak_ of the few remaining Ca^2+^ transients. **(C)** Ca^2+^ transients depend on presynaptic voltage-gated Ca^2+^ channel activity necessary for neurotransmitter release. Treatment with ω-CTx (0.1–0.2 μM; 1 h) reduced F_S_ by 62 ± 7% (*n* = 30; *N* = 2) without affecting A_S,Peak_. In a separate explant, the response of 15 neurons to DMPP (10 μM) applied as in Figure 1 was unaffected by ω-CTx treatment (inset). **(D–F)** Most Ca^2+^ transients depend on nAChR activation. **(D)** Hex (100–200 μM, 15 min) reduced F_S_ by 42 ± 9% (*n* = 52; *N* = 3) without affecting A_S,Peak_. **(E)** Combining 100 μM mHex with 100 μM dTC (Hex/dTC, 15 min) had nearly identical effects, reducing F_S_ by 51 ± 13% (*n* = 29; *N* = 1) without affecting A_S,Peak_. **(F)** Treatment with SR16584 (40 μM, 15 min) to selectively target α3β4*-nAChRs, potently inhibited the MG neuron Ca^2+^ transients reducing F_S_ to zero in 35 of 50 neurons (85 ± 5% reduction overall; *n* = 50, *N* = 3) and having no effect on residual A_S,Peak_ in 7 of 15 neurons while reducing A_S,Peak_ in the remaining 8 neurons by 64%. **(G,H)** Most Ca^2+^ transients also depend on 5-HT_3_R activation. Ondansetron (20 μM; 10–20 min) reduced F_*S*_ by 70 ± 10% and A_S,Peak_ by 54 ± 4% (*n* = 51; *N* = 4) while in one experiment LY278584 (10 μM; 20 min) reduced F_S_ by 67 ± 15% while nominally reducing A_S,Peak_ by 14 ± 25% (*n* = 15).

Pharmacological tests were conducted to identify cellular processes underlying the spontaneous Ca^2+^ transients in ChAT^+^/GCaMP6f^+^ MG neurons (Figure 3). A requirement for action potentials was assessed first, initially using TTX to inhibit voltage-gated Na^+^ channel-dependent action potentials (Figure 3A). In paired comparisons made before and after treatment, TTX application (2 μM, 15 min) reduced the frequency of Ca^2+^ transients (F_S_) by 61% without detectably affecting the amplitudes (A_S,Peak_) or kinetics (T_R,1/2_ and T_D,1/2_) of residual events (data not shown). The ability of TTX to reduce Ca^2+^ transient F_S_ without affecting A_S,Peak_ would be expected if the toxin blocked TTX-sensitive Na^+^ channels on presynaptic axonal inputs to cholinergic MG neurons while having less of an effect on somatic Na^+^ channels, some of which are known to be TTX-resistant and sustain Ca^2+^ influx (Rugiero et al., 2003; Mao, 2006) an outcome that would leave A_S,Peak_ unaffected. If such a mechanism applied, blocking both TTX-sensitive and -resistant Na^+^ channel dependent impulses would be expected to drastically reduce both F_S_ and A_S,Peak_. The local anesthetic lidocaine (Lido) is a pan-selective Na^+^ channel inhibitor that blocks TTX-resistant Na currents (Scholz et al., 1998) and combining the two inhibitors (TTX/Lido) to target TTX-sensitive and -resistant Na^+^ channels resulted in a nearly complete blockade of Ca^2+^ transients, lowering F_S_ by 96% and reducing A_S,Peak_ of the few remaining Ca^2+^ transients by 60% (Figure 3B). Taken together, these findings indicate that the spontaneous Ca^2+^ transients in cholinergic MG neurons require Na^+^ channel dependent impulses occurring in axonal presynaptic inputs that lead to action potentials in postsynaptic somas.

### Spontaneous Ca^2+^ Transients in Cholinergic MG Neurons Reflect Ongoing Synaptic Activity

The TTX experiments suggest that the abundant spontaneous Ca^2+^ transients observed in cholinergic MG neuron somas arise from synaptic interactions that are preserved in intact *ex vivo* preparations. In stripped preparations, by contrast, intracellular recordings from MG neurons reveal limited instances of spontaneous excitatory postsynaptic potentials (EPSPs) (Fung et al., 2018). To determine whether the spontaneous Ca^2+^ transients in ChAT^+^/GCaMP6f^+^ MG neurons result from synaptic activity, mechanisms supporting both pre- and postsynaptic function were probed. A requirement for presynaptic activity was assessed using ω-conotoxin-MVIIC (ω-CTx) a neurotoxin that targets and blocks voltage-gated P/Q- and N-type Ca^2+^ channels (Ca_V_2.1 and Ca_V_2.2, respectively) that are prevalent on presynaptic terminals (Wu and Saggau, 1995; McDonough et al., 1996) and responsible for initiating neurotransmitter release (Mochida, 2018). Consistent with an all-or-none presynaptic action, ω-CTx reduced ChAT^+^/GCaMP6f^+^ MG neuron Ca^2+^ transient F_S_ by 63% and had no effect on Ca^2+^ transient A_S,Peak_ relative to the same neurons tested prior to toxin treatment (Figure 3C). The action of ω-CTx was specific for presynaptic Ca^2+^ channels in the sense that the same toxin incubation paradigm did not alter the DMPP-induced response in somas (as in Figure 1) where L-type Ca^2+^ channels (Ca_V_1.1–1.4) are prominent (Hell et al., 1993; Simon et al., 2003) and expected to be activated by DMPP-induced depolarization. Because most cholinergic MG neurons display robust responses to nAChR activation (Figure 1) and nAChRs are known to mediate fast transmission in myenteric ganglia (Galligan and North, 2004; Gwynne and Bornstein, 2007; Foong et al., 2015) initial tests to determine if postsynaptic ionotropic receptor function was required for the Ca^2+^ transients were conducted using nAChR inhibitors (Figures 3D–F). Treatment with Hex alone or with Hex in combination with dTC (Hex/dTC) reduced Ca^2+^ transient F_S_ by 42 or 47%, respectively, in both cases without affecting A_S,Peak_ of residual events. The α3β4^∗^-AChR selective antagonist SR16584 strongly inhibited the Ca^2+^ transients, reducing F_S_ to zero in most cases and by 85% overall, while reducing A_S,Peak_ in the few residual events by 38% overall. The ability of nAChR antagonists to reduce F_S_ while having no or, in the case of SR16584, a lesser effect on A_S,Peak_ indicates that nAChR inhibition causes a large fraction of the Ca^2+^ transients to become undetectable, consistent with the ability of the antagonists to drastically inhibit the DMPP response. Taken together the use of antagonists to probe pre- and postsynaptic functions indicate that the Ca^2+^ transients in ChAT^+^/GCaMP6f^+^ MG neurons arise from presynaptic activity, and that a substantial fraction are dependent on postsynaptic α3β4^∗^-nAChR activation.

Because Hex/dTC spared many and SR16584 spared some of the Ca transients (Figures 3E, F) the possibility was examined that the events unaffected by the nAChR antagonists reflect synaptic activation of receptors other than nAChRs. Since functional 5-HT_3_Rs were detected on cholinergic MG neurons (Figure 1) their relevance to the Ca^2+^ transients was explored using selective antagonists (Figures 3G,H). When compared to the same neurons assayed before and after antagonist treatment, application of Ondansetron reduced F_S_ by 70% and in one experiment LY278584 did so by 67%. While the nAChR antagonists failed to affect amplitude, Ondansetron also reduced A_S,Peak_ of residual Ca^2+^ transients doing so by 54% whereas LY278584 nominally reduced A_S,Peak_ by 14% in the few remaining unaffected neurons. The different effects of Ondansetron and LY278584 on A_s,Peak_ may reflect different potencies of these antagonists. Nevertheless, the ability of both 5-HT_3_R antagonists to drastically reduce F_S_ and that of Ondansetron to also reduce A_S,Peak_ is consistent with postsynaptic 5-HT_3_R inhibition where most Ca^2+^ transients disappear and a few persist at reduced size. Taken together, the preceding results with ω-CTx and ionotropic serotonin and acetylcholine receptor antagonists indicate that the Ca^2+^ transients routinely observed in cholinergic MG neurons in unstripped, intact colon explants reflect a dependence on synaptic transmission mediated by nAChRs, particularly α3β4^∗^-nAChRs, by 5-HT_3_Rs, or by combined activation of both receptor types.

### The nAChR and 5-HT_3_R Dependent Ca^2+^ Transients in Cholinergic MG Neurons Reflect Activation of Poly- and Mono-Synaptic Circuits Mediated by Either and Both Receptor Types

**FIGURE 4:**
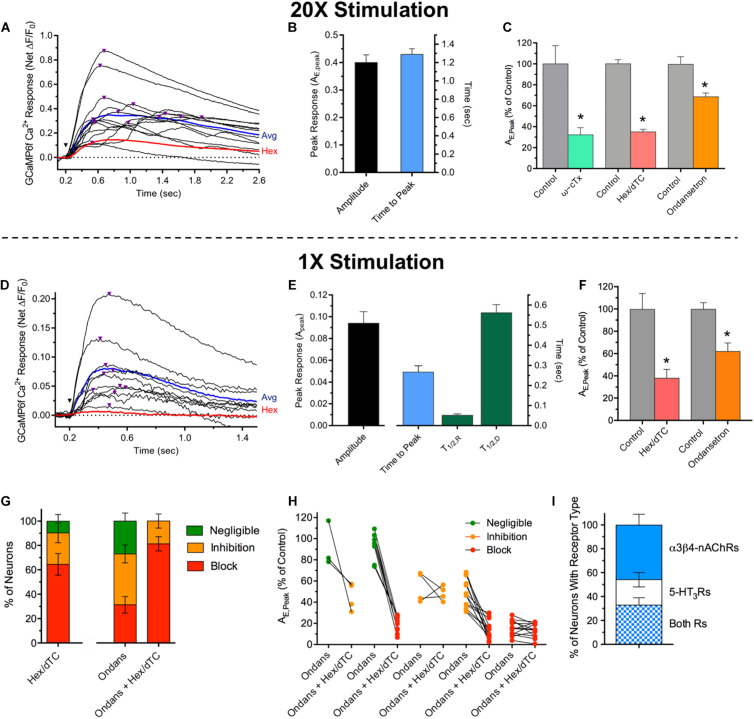
Ca^2+^ responses evoked by MG connective stimulation depend on presynaptic voltage-gated Ca^2+^ channel activity and activation of postsynaptic nAChRs or/and 5-HT_3_Rs. **(A)** Time course of Ca^2+^ fluorescence changes in 13 neuron somas (black traces) evoked by a train of 20 stimuli (1500 μA, 10 Hz; 20× stimulation) delivered at the inverted black triangle. Purple triangles above the traces depict the timing of peak responses (A_E,Peak_). The response average from these neurons and that of the same neurons after incubation with Hex (100 μM, 15 min) are depicted by the blue and red traces, respectively. **(B)** Summary of responses to 20× stimulation. 20× stimulation evoked an average peak responses of A_E,peak_ = 0.41 ± 0.02 with an average time to peak response of 1.29 ± 0.06 s (*n* = 175, *N* = 4). **(C)** 20× response pharmacology. A_E,peak_ values for neurons treated with antagonists are presented relative to those obtained from the same neurons assayed before incubation in drug. Treatment with ω-CTx (0.1–0.2 μM; 60 min) to block presynaptic voltage-gated Ca^2+^ channels (green bar), Hex/dTC (100 μM each; 15 min) to block nAChRs (salmon bar), or Ondansetron (20 μM; 15 min) to block 5-HT_3_Rs (orange bar) significantly reduced A_E,Peak_ to levels that were 32 ± 7, 35 ± 3, or 68 ± 4%, respectively, of those obtained from the same neurons assessed before antagonist application (gray bars, Control; *p < 0.05* for each). For ω-CTx, Hex, and Ondansetron the corresponding replicates were *n* = 15, 168, and 196 and *N* = 1, 4, and 3. **(D)** Time course of Ca^2+^ fluorescence changes in nine neuron somas (black traces) evoked by a single 1500 μA stimulus (1× stimulation) delivered at the inverted black triangle. Purple triangles above the traces depict the timing of peak responses (A_E,Peak_). The response average from these neurons and that of the same neurons after incubation with Hex (100 μM; 15 min) are depicted by the blue and red traces, respectively. **(E)** Summary of responses to 1× stimulation. *Left:* A_E,Peak_ values obtained in response to 1× stimulation (0.091 ± 0.013, *n* = 42; *N* = 2) were indistinguishable from those associated with spontaneous Ca^2+^ transients (Figure 2; *p* > 0.05). *Right*: The latency to A_E,Peak_ was 0.268 ± 0.031 s (*n* = 42; *N* = 2). The responses to 1× stimulation displayed rise and decay time kinetic parameters (T_R,1/2_ = 0.053 ± 0.005 s and T_D,1/2_ = 0.563 ± 0.039 s; *n* = 30; *N* = 2) values not significantly different from those for the spontaneous Ca^2+^ transients (Figure 2; *p* > 0.05). **(F)** Pharmacology. Treatment with Hex or Hex/dTC (Hex/dTC, 100 μM each; 15 min) to block nAChRs (salmon bar) significantly reduced A_E,Peak_ to 38 ± 8% (*n* = 31; *N* = 2) while application of Ondansetron to block 5-HT_3_Rs reduced A_E,Peak_ to 62 ± 7% (*n* = 48; *N* = 3) both relative to the same neurons assessed before antagonist application (gray bars, Control). **(G)** Differential effects of antagonists on A_E,Peak_ responses in subsets of MG neurons. Antagonist effects on Ca^2+^ responses to 1× stimulation between treated and control neuron pairs were subdivided as block (≥70% reduction of A_E,Peak_, red) inhibition (69–30% reduction, orange) or negligible (≤29% reduction, green) and expressed as the *per cent* of neurons in each category (*n* = 48, *N* = 3). *Left:* effect of Hex/dTC. *Middle and right:* effect of Ondansetron, followed in the same cells by Ondansetron *and* Hex/dTC. **(H)** Transition of individual neurons in **(G)** that were unaffected (green) or inhibited (orange) by Ondansetron, to inhibited or blocked (red) following treatment with Ondansetron *and* Hex/dTC. Note that some neurons inhibited or blocked by Ondansetron remained so after treatment with both antagonists. **(I)** Proximal colon MG neurons can express functional α3*β4-nAChRs but not 5-HT_3_Rs, 5-HT_3_Rs but not α3*β4-nAChRs, or both receptor types. DMPP or 5-HT were applied sequentially to MG neurons within a given field to activate α3*β4-nAChRs or 5-HT_3_Rs, respectively. Results are plotted as the *per cent* of responsive neurons (*n* = 364, *N* = 4) that were sensitive to DMPP but not 5-HT (46 ± 9% α3*β4-nAChR only) to 5-HT but not DMPP (21 ± 6% 5-HT_3_R only) and to both agonists (33 ± 6% both receptor types).

To determine if evoked Ca^2+^ responses and the spontaneous Ca^2+^ transients share similar synaptic mechanisms, and whether the evoked responses are consistent with functional transmission mediated by poly- and/or monosynaptic inputs, current pulses were applied to MG connectives (Figure 4). Single stimuli or stimulus trains (1500 μA, 100–200 μs; 1× or 20×) delivered to connectives 5–6 mm oral to a MG reliably evoked sharp onset ΔF/F_0_ Ca^2+^ increases in cholinergic MG neurons. In all ChAT^+^/GCaMP6f^+^ colon explants 20× stimulation evoked such events, doing so in 97% of randomly selected ChAT^+^/GCaMP6f^+^ neurons with an average evoked net peak response (A_E,Peak_) of 0.41 (Figures 4A, B). Consistent with expectations that inputs to MG neurons would converge and responses to stimulus trains would summate over time, the A_E,Peak_ responses to 20× stimulation (Figure 4C) were four times larger than the spontaneous Ca^2+^ transients (Figure 2) and displayed wide variability in times-to-peak (ranging from 0.3 to 3.7 s). In order to better resolve individual evoked synaptic responses, single stimuli were applied (Figures 4D–G). In 71% of ChAT^+^/GCaMP6f^+^ neurons 1× stimulation evoked abrupt increases in Ca^2+^ fluorescence that closely resembled the spontaneous Ca^2+^ transients. In particular, responses evoked by 1× stimulation having fast rise, slow decay changes in Ca^2+^ fluorescence had a mean peak amplitude of A_E,Peak_ = 0.09, a value not significantly different from that of spontaneous Ca^2+^ transients (A_S,Peak_ = 0.11, Figure 2; *p > 0.05*) and temporally isolated responses displayed respective T_R,1/2_ and T_D,1/2_ values of 53 and 563 ms, that were also not significantly different from those of the spontaneous Ca^2+^ transients (Figure 2; 67 and 546 ms; *p* > 0.05 for both).

As was the case for the spontaneous Ca^2+^ transients, the responses evoked by MG connective stimulation, required synaptic transmission. *First*, like the Ca^2+^ transients, the evoked A_E,Peak_ responses were sensitive to inhibition of presynaptic Ca^2+^ channels, with ω-CTx reducing the responses to 20× stimulation by 68% compared to those of the same neurons tested prior to toxin treatment (Figure 4C). *Second*, MG neuron responses evoked by connective stimulation were reduced after treatment with nAChR or/and 5-HT_3_R antagonists. In both ChAT^+^/GCaMP6f^+^ and E2a^+^/GCaMP6s^+^ neurons, average A_E,Peak_ response values to 20× stimulation were reduced by 65% compared to paired controls following nAChR inhibition with Hex/dTC, while 5-HT_3_R inhibition with Ondansetron caused a smaller 32% reduction (Figure 4C). Average paired response values to 1× stimulation were similarly affected with nAChR inhibition causing a more potent 62% reduction in A_E,Peak_ compared to the 32% reduction following 5-HT_3_R inhibition (Figures 4D, F). Inspection of individual response pairs revealed that antagonizing a single receptor type resulted in non-uniform inhibition with stimulus evoked responses blocked in some neuron pairs (i.e., A_E,Peak_ ≈ 0) and inhibited, or unaffected in others (Figure 4G). Applying these criteria to 1× stimulation reveals that treatment with Hex/dTC blocked evoked responses in 65% of MG neuron pairs, inhibited responses in 25% and had little or no detectable effect in 10%. By contrast, Ondansetron blocked A_E,Peak_ responses evoked by 1× stimulation in only 31% of MG neuron pairs while inhibiting responses in 42% and having no detectable effect in 27%. Subsequent co-application of both Ondansetron and Hex/dTC to the same neurons initially exposed to Ondansetron alone altered the distribution, increasing response block to 81% of MG neuron pairs, with 19% showing mild inhibition and none unaffected (Figure 4G). The 50% increase in neurons showing response block is consistent with the additional blockade of a large fraction of neurons expressing nAChRs and supports the conclusion that most MG neurons display stimulus evoked synaptic Ca^2+^ responses that are mediated by nAChRs, with a subpopulation displaying responses mediated by 5-HT_3_Rs. In addition, the transition of individual neurons from inhibited and unaffected to blocked following the application of both antagonists indicates that evoked Ca^2+^ responses are mediated by both nAChRs and 5-HT_3_Rs in a subset of MG neurons (Figure 4H). Consistent with this idea, puffer application of DMPP then 5-HT or *vice versa* demonstrated that MG neurons express functional receptors of either or both type (Figure 4I). Thus, for proximal colon MG neurons sensitive to either agonist, 46% displayed A_A,Peak_ Ca^2+^ responses mediated solely by nAChRs, 21% displayed responses mediated solely by 5-HT_3_Rs, and 33% had responses mediated by both receptor types. In accord with the functional relevance of α3β4^∗^-nAChRs and 5-HT_3_Rs demonstrated by the preceding results, RNAscope *in situ* hybridization studies revealed mRNA for α3-nAChR subunit in MG neurons, and immuno-labeling experiments revealed punctate 5-HT immunoreactivity on MG neurons consistent with a presynaptic localization (Supplementary Figures 2A, B).

**FIGURE 5:**
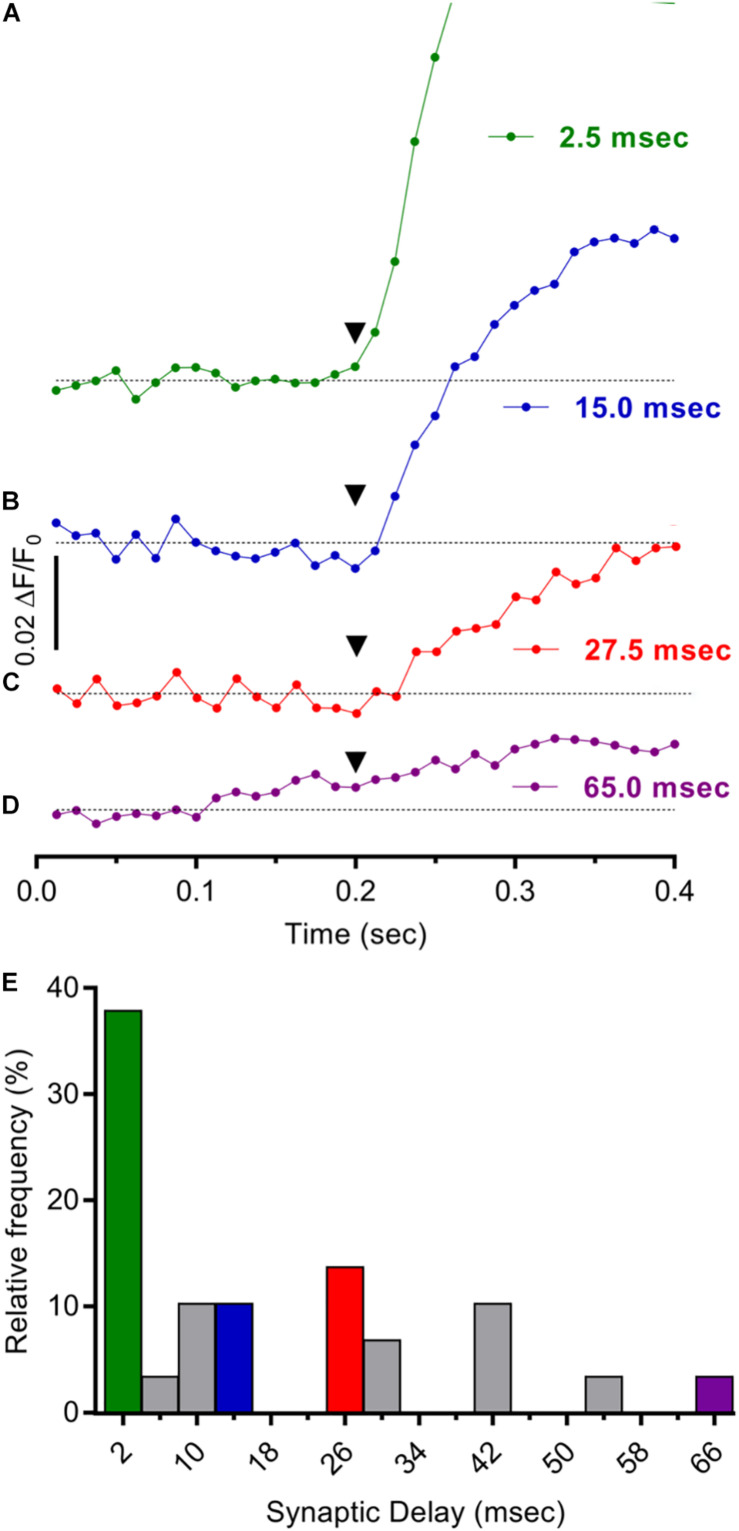
Mono- and poly-synaptic pathways underlie stimulus evoked Ca^2+^ responses in cholinergic MG neurons. **(A–D)**. Individual records depicting expanded time courses of Ca^2+^ fluorescence changes sampled at 80 Hz in four GCaMP6f^+^/ChAT^+^ neurons from a MG following one 1500 μA stimulus. The time elapsed from stimulus application (inverted black triangles at 200 ms) to response onset [2× SD of pre-stimulus ΔF/F_0_ (response latency)] was determined for each neuron as described in the text. Synaptic delay times were calculated by subtracting the time required for impulse conduction to the recording site [based on a conduction velocity of 0.55 mm/ms (Stebbing and Bornstein, 1996)] from the response latency and were on average 17.7 ± 3.3 ms (*n* = 29; *N* = 2) and 1.5, 15.0, 27.5, and 65.0 ms, respectively, for the neurons depicted in **(A–D)**. **(E)** Consistent with the prevalence of monosynaptic inputs, synaptic delay times <4 ms were most common. Histogram depicts the frequency distribution of synaptic delay times for 29 neurons with colors matching records in **(A–D)**. Note that the largest fraction of the entries (38%) correspond to synaptic delay times <4 ms while only 10, 7, and 3% correspond to 15, 27, and 65 ms synaptic delay times, respectively.

Response latencies were next assessed to determine if the ΔF/F_0_ Ca^2+^ responses evoked in cholinergic MG neurons by 1× connective stimulation arise from activation of mono- or poly-synaptic circuits, or both (Figure 5). For each stimulus, the response latency was measured as the time elapsed between its application and a ΔF/F_0_ increase 2–4× SD above pre-stimulus levels. Using this criterion, the average response latency to 1× stimulation was 28 ms (*n* = 29). To estimate synaptic delay times, the time required for action potentials initiated in presynaptic axons to travel from the site of the stimulating electrode to the center of the microscope field (5–6 mm) was calculated from the conduction velocity for longitudinally projecting mouse myenteric axons (0.55 mm/ms) (Stebbing and Bornstein, 1996) and subtracted from overall response latency values. Synaptic delay times obtained in this fashion had a mean value of 17.7 ± 3.3 ms, with individual times broadly distributed from 1.5 to 65.0 ms (e.g., Figures 5A–D). A previous estimate of the delay time for fast synapses impinging on MG neurons was 1.9 ms (Stebbing and Bornstein, 1996) a value consistent with measured monosynaptic delays at the neuromuscular junction (0.5–4.0 ms) (Katz and Miledi, 1965) and central synapses (1.0–3.0 ms) (Goyal and Chaudhury, 2013). The range of synaptic delays estimated here indicates that both mono and poly-synaptic inputs underlie responses in cholinergic MG neurons evoked by single longitudinal input stimuli. Because nearly 40% of synaptic delays ranged from 1.5 to 4.0 ms (Figure 5E) however, monosynaptic inputs can account for the largest fraction of the responses.

### Modulation of Spontaneously Active Myenteric Circuits

**FIGURE 6:**
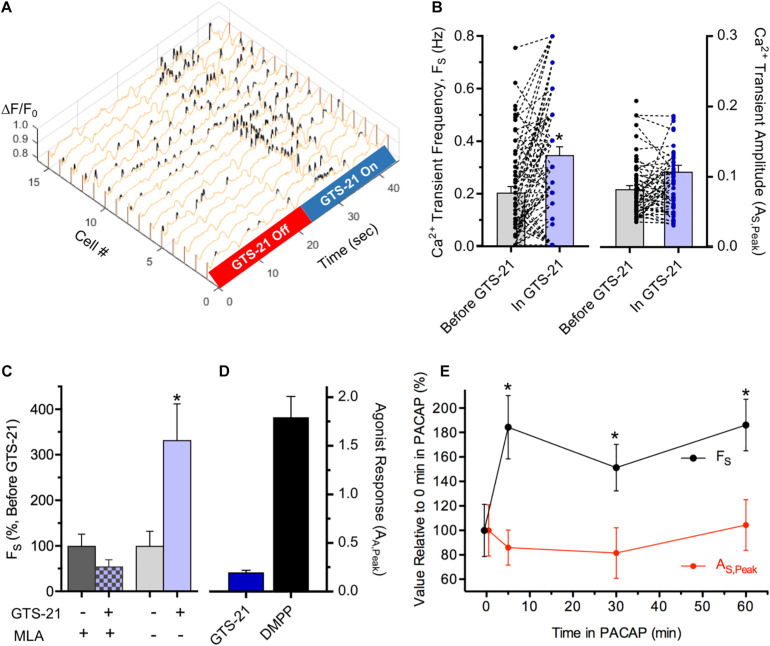
Modulation of synapse-dependent spontaneous Ca^2+^ transients. **(A–D)** Modulation by α7-nAChR activation. **(A)** Ca^2+^ transient frequency (F_S_) in cholinergic myenteric neurons increases during perfusion with GTS-21, an α7-nAChR selective agonist. Exemplar 3D plot showing Ca^2+^ ΔF/F_0_ transients in 15 neurons before and during puffer application of GTS-21 (10 μM; 5 psi). **(B)** α7-nAChR activation by GTS-21 selectively increases Ca^2+^ transient frequency. Data presented as in Figure 3. Overall, GTS-21 increased Ca^2+^ transient F_S_ by 126 ± 25% (left) in neurons exposed to GTS-21 compared to F_S_ in the same neurons prior to GTS-21 exposure (*n* = 72; *N* = 5), whereas GTS-21 exposure failed to detectably change Ca^2+^ transient A_S,Peak_ (right; *p = 0.24*). **(C)** The α7-nAChR selective antagonist MLA blocks GTS-21 induced modulation. Pre-incubation with MLA (50 nM, 1 h) had no detectable effect on F_S_, but blocked the ability of GTS-21 to increase F_S_ (left; *n* = 16; *N* = 2). Prior to MLA application, GTS-21 increased F_S_ by 232 ± 90% in the same neurons (right). **(D)** Indicative of a presynaptic site of action, GTS-21 induced very small peak increases in ΔF/F_0_ (A_*E,Peak*_) (*n* = 56; *N* = 4) compared to DMPP responses assessed in the same explants (*n* = 30). **(E)** Ca^2+^ transient modulation by PACAP. Ca^2+^ transient frequency (F_S_) and amplitude (A_S,Peak_) are plotted versus time in 100 nM PACAP as a *per cent* of their values before PACAP exposure (17–38 transients from 15 neurons). Chamber perfusion was turned off during PACAP exposure. Note the persistent ≈90% elevation of F_S_ after 5, 30, and 60 min in PACAP (*p* < 0.05 for each) without a detectable change in A_S,Peak_.

Activation of ionotropic α7-nAChRs and neuropeptide-activated G-protein coupled receptors (GPCRs) can potently modulate synaptic function. At central and autonomic synapses where transmission is mediated by non-N-Methyl-D-aspartate glutamate receptors (non-NMDARs) and α3β4^∗^-nAChRs, respectively, nicotine acting *via* highly Ca^2+^ permeable presynaptic α7-nAChRs (Séguéla et al., 1993) elevates Ca^2+^ in presynaptic terminals, thereby enhancing neurotransmitter release and increasing the frequency (but not the amplitude) of excitatory postsynaptic currents (EPSCs) (McGehee et al., 1995; Gray et al., 1996; McGehee and Role, 1996). Because *in situ* hybridization studies demonstrated the presence of α7-nAChR subunit transcripts in MG tissue (Supplementary Figure 2C) a modulatory role for α7-nAChRs at synapses on cholinergic MG neurons was tested (Figure 6). To do so, ChAT^+^/GCaMP6f^+^ colon explants were challenged with the α7-nAChR specific agonist, 3-(2,4-dimethoxybenzylidene)-anabaseine dihydrochloride (GTS-21) (Briggs et al., 1997; Meyer et al., 1997; Nai et al., 2003) during the acquisition of spontaneous Ca^2+^ transients (Figure 6A). Consistent with a presynaptic site of action, GTS-21 application increased the frequency of GCaMP6f-mediated Ca^2+^ transients (F_S_) by 125% when compared to the same neurons assayed prior to treatment (Figure 6B) and did so without detectably altering their amplitudes (A_S,Peak_). The increase in F_S_ was short-lived, being evident only within the period of GTS-21 application. GTS-21 acted specifically on α7-nAChRs because it failed to increase F_S_ when colon explants were pre-treated with methyllycaconitine (MLA) an α7-selective antagonist (Ward et al., 1990; Figure 6C). The ability of GTS-21 to modulate Ca^2+^ transient F_S_ was consistent with a presynaptic site of action, specifically by targeting α7-nAChRs present on presynaptic inputs to cholinergic MG neurons rather than on somatic α7-nAChRs. *First*, although α7-nAChRs have high permeability to Ca^2+^, displaying a Ca^2+^/Na^+^ permeability ratio of ≈20 (Séguéla et al., 1993) they desensitize rapidly and have extremely brief channel open times (McNerney et al., 2000; Nai et al., 2003; Corradi and Bouzat, 2016) making it unlikely that they would produce a depolarization sufficient to evoke somatic signals detectable by GCaMP6f. *Second*, in accord with the failure of GTS-21 to increase A_S,Peak_ as seen here, or EPSC amplitudes as reported previously (McGehee et al., 1995) GTS-21 typically evoked only small increases in net ΔF/F_0_ whereas neurons from the same explants responded to DMPP with robust responses (Figure 6D).

Pituitary adenylate cyclase activating polypeptide (PACAP) and vasoactive intestinal peptide (VIP) belong to a family of homologous neuropeptides that bind to specific GPCRs and both have broad expression and functional relevance in the GI tract (reviewed by Reglodi et al., 2018). Relevant to autonomic synapses, PACAP is released from preganglionic terminals, activates high-affinity PACAP GPCRs (PAC_1_Rs) and enhances short-term nAChR mediated synaptic function within minutes (Pugh et al., 2009; Jayakar et al., 2014). Because PACAP and PAC_1_Rs are present in MP neurons and fibers (Portbury et al., 1995; Miampamba et al., 2002) the ability of PACAP to modulate spontaneous synaptic Ca^2+^ transients in cholinergic MG neurons was tested (Figure 6E). After 5 min exposure to PACAP, Ca^2+^ transient F_S_ nearly doubled. Unlike the short-lived modulation caused by α7-nAChR activation, however, the increase in F_S_ caused by PACAP remained elevated, compared to the same neurons assessed prior to PACAP application, for at least 60 min as anticipated for a GPCR mediated regulation. Despite its ability to enhance F_S_, PACAP treatment had no detectable effect on Ca^2+^ transient amplitudes throughout the 60 min test period.

## Discussion

Targeted GCaMP6f Ca^2+^ imaging reveals abundant, diverse, and modifiable synaptic activity displayed by cholinergic MG neurons in the intact mouse colon. GCaMP6f is well suited for detecting evoked and spontaneous neuronal activity in a multi-layered tissue such as the mouse intestine because of its high resting fluorescence, Ca^2+^ sensitivity, and kinetic properties (Chen et al., 2013) and because a mouse strain containing a floxed-STOP GCaMP6f construct is commercially available. While new and improved GECIs such as GCaMP8f and XCaMP-Gf are being developed, side-by-side comparisons of sensitivity and kinetics indicate that GCaMP6f detects single neuron spikes with comparable fidelity and linearity (Inoue et al., 2019).^1^

Puffer application of DMPP evoked robust GCaMP6f Ca^2+^ responses in all proximal mouse colon segments tested (Figures 1A–D) doing so in 69% of cholinergic (i.e., ChAT^+^/GCaMP6f^+^) MG neurons. Pharmacological tests indicated that the DMPP responses were reduced by ≈70% after treatment with the generic nAChR inhibitor, Hex or with the α3β4^∗^-specific nAChR inhibitor, SR16584 and to a somewhat greater extent (≈90%) after treatment with the generic nAChR inhibitor dTC (alone or in combination with Hex). These results indicate that α3β4^∗^-nAChRs represent the bulk of nAChRs on cholinergic MG neurons in the adult mouse colon, consistent with previous electrophysiological studies underscoring the importance of cholinergic signaling *via* ganglionic α3β4^∗^-nAChRs in mouse and guinea pig MP neurons (Nurgali et al., 2003; Galligan and North, 2004; Gwynne and Bornstein, 2007). A previous study in mouse gut where α3 and β4-nAChR subunits predominate, showed that SR16584 inhibited the DMPP response of adult MG neurons in duodenum to a lesser extent than did Hex (Foong et al., 2015) suggesting that the distribution of α3β4^∗^-nAChRs on the neurons differs between small and large intestine. Other electrophysiological studies have shown that 5-HT induces excitatory responses in guinea pig enteric neurons that are indicative of permeability to cations including Ca^2+^ and mediated by 5-HT_3_Rs (Mawe et al., 1986; Derkach et al., 1989; Yang, 1990; Galligan et al., 2000). Consistent with these observations we found that 5-HT application evoked large Ca^2+^ ΔF/F_0_ responses in 62% cholinergic MG neurons that were attributable to 5-HT_3_Rs (Figures 1E–H). In proximal colon explants from E2a^+^/GCaMP6s^+^ mice, sequential application of DMPP and 5-HT revealed that one third of MG neurons as a whole co-express functional nAChRs and 5-HT_3_Rs. Consistent with previous studies indicating that ≈50% of neurons in mouse proximal colon are ChAT+ (Li et al., 2019b) our recent findings indicate that ≈60% of mouse proximal colon MG neurons are ChAT+ (Nestor-Kalinoski et al., in review) making it likely that α3β4^∗^-nAChRs and 5-HT_3_Rs are co-expressed in a substantial fraction of cholinergic MG neurons.

In addition to sensitivity to DMPP and 5-HT, cholinergic MG neurons in nearly all intact unstimulated colon segments displayed spontaneous activity in the form of Ca^2+^ transients (Figure 2). Consistent with their identification as discrete synapse-driven events, the Ca^2+^ transients featured rapid rise and slower decay times (T_R,1/2_ and T_D,1/2_, respectively). While the observed T_R,1/2_ (67 ms) is consistent with previous values obtained for GCaMP6f-mediated Ca^2+^ transients induced by single action potentials in mammalian cortical neuron somas, the observed T_D,1/2_ (546 ms) is 2.7–3.8 times longer (Chen et al., 2013). Two factors that would reduce the rate of Ca^2+^ extrusion and hence explain the larger T_D,1/2_ associated with MG neurons are a higher endogenous buffering capacity and, because extrusion scales with the membrane surface area (Cornelisse et al., 2007; Badura et al., 2014) a smaller surface to volume ratio (SVR). Indeed, soma size considerations predict that MG neurons will have a 2.5× smaller SVR relative to cortical neurons (≈25 μm and ≈10 μm soma diameters, respectively). Moreover, Ca^2+^ buffering capacity and cell geometry have been shown previously to influence the decay of Ca^2+^ signals induced by single action potentials in dendrites where a threefold higher endogenous buffering capacity and > twofold smaller SVR in large diameter dendritic shafts compared to smaller spines both contribute to the larger T_D,1/2_ of Ca^2+^ decay in dendritic shafts (Cornelisse et al., 2007). Taken together these considerations indicate that GCaMP6f is sufficiently sensitive to report Ca^2+^ fluctuations resulting from single MG neuron action potentials that trigger Ca^2+^ influx *via* voltage-gated Ca^2+^ channels. GCaMP6f may also be able to detect subthreshold synaptic activity, as was previously shown in hippocampal and somatosensory neurons (Peron et al., 2015; Li et al., 2019a). Because a major fraction of fast excitatory synaptic transmission between enteric neurons is mediated by nAChRs (Galligan and North, 2004; Gwynne and Bornstein, 2007; Foong et al., 2015) which are highly permeable to Ca^2+^ as well as Na^+^ (Fucile, 2004) the Ca^2+^ transients may also reflect sub-threshold EPSPs that result from synaptic activation of nAChRs and other Ca^2+^ permeable ionotropic receptors.

A series of pharmacological experiments with TTX and Lido to block neuronal action potentials, ω-CTx to block presynaptic Ca^2+^ channel dependent neurotransmitter release, and postsynaptic receptor antagonists revealed that the spontaneous Ca^2+^ transients arose from synaptic activity intrinsic to the colon segment (Figure 3). We surmise that many of the synaptic interactions present *in vivo* are preserved in the intact colon segments used here, thereby increasing the likelihood of spontaneous synaptic events. In accord with this consideration and the importance of nAChRs in mediating fast synaptic transmission to MG neurons, nAChR antagonists (Hex, Hex/dTC, and SR16584) all reduced Ca^2+^ transient frequency (F_S_) and did so without affecting the amplitude (A_S,Peak_) of the spared events except in rare cases following SR16584 treatment. As mentioned in section “Results,” these findings are consistent with an “all-or-none” phenomenon where the antagonists drive synaptic responses below the GCaMP6f detection levels, but for some events the nAChR antagonists have no effect, leaving their amplitudes unchanged suggesting they are mediated by other receptor types. In this regard, 5-HT_3_Rs were also found to contribute to the spontaneous Ca^2+^ transients because selective antagonists (LY278584 and Ondansetron) both reduced F_S_. Interestingly, Ondansetron also reduced A_S,Peak_ suggesting it induces an incomplete block of some 5-HT_3_Rs, or reduces the amplitude of transients mediated by 5-HT_3_Rs and other receptor types. It is worth noting that some of the spontaneous Ca^2+^ transients may represent subthreshold Ca^2+^ entry *via* nAChRs and 5-HT3Rs since both display significant Ca^2+^ permeability (Yang, 1990; Fucile, 2004).

Connective stimulation revealed further evidence for MG synaptic transmission mediated by two postsynaptic receptor types (Figure 4). 20× connective stimulation evoked synaptic A_E,Peak_ responses in cholinergic MG neurons, requiring ω-Ctx sensitive presynaptic Ca^2+^ channels and depending, in most cases, on postsynaptic nAChRs or/and 5-HT_3_Rs. Experiments using 1× stimulation confirmed these observations, revealing a similar dependence on nAChRs, or/and 5-HT_3_Rs. Moreover, the Ca^2+^ responses evoked by 1× stimulation displayed amplitudes, half-rise and half-decay times that were indistinguishable from those of the spontaneous Ca^2+^ transients (compare Figure 4E with Figures 2C, D) supporting the conclusion that the Ca^2+^ transients arise from ongoing synaptic activity generated by nAChRs, specifically α3β4^∗^-nAChRs. Since Hex/dTC blocked A_E,Peak_ responses evoked by 1× connective stimulation in a significantly larger fraction of MG neurons than did Ondansetron (65 versus 31%, *p* < 0.05) our results indicate that, while 5-HT_3_Rs participate, nAChRs and specifically α3β4^∗^-nAChRs, play a predominant role in synaptic responses to connective stimulation. In addition, the migration of inhibited and unaffected MG neurons following Ondansetron treatment to the blocked population after exposure to *both* Ondansetron and Hex/dTC suggests that both nAChRs and 5-HT_3_Rs participate in stimulus evoked responses in a subset of MG neurons. Results from agonist co-application experiments support this interpretation, as do previous studies in guinea pig and rat demonstrating that ganglionic connective stimulation evokes fast EPSPs mediated solely by nAChRs in 25–100% of MG neurons, and by both nAChRs and 5-HT_3_Rs in smaller sub-populations (Browning and Lees, 1996; Zhou and Galligan, 1999).

Because a few evoked events survive after treatment with nAChR or/and 5-HT_3_R antagonists, our findings further indicate that other postsynaptic ionotropic receptors contribute to the Ca^2+^ transients in mouse colon. Guinea pig sensory MG neurons express functional ionotropic γ-aminobutyric acid receptors; GABA application produces excitatory inward currents (reversing polarity at +4 mV) that are blocked by the GABA_A_R antagonist bicuculline (Zhou and Galligan, 2000). Similarly, we found that a minor fraction (≈15%) of mouse cholinergic MG neurons displayed Ca^2+^ increases in response to puffer-applied GABA that were inhibited by bicuculline (Supplementary Figures 3A, B). It is unlikely, however, that GAB_A_Rs underlie or influence either the spontaneous or stimulus evoked Ca^2+^ transients because bicuculline failed to affect the amplitude or frequency of the spontaneous transients, or the amplitude of responses evoked by connective stimulation (Supplementary Figures 3C, D). Mouse MG neurons also display Ca^2+^ responses to glutamate detected by GCaMP3 that are mediated by ionotropic NMDARs and not by α-amino-3-hydroxy-5-methyl-4-isoxazole propionic acid receptors (AMPARs). Despite the presence of functional NMDARs, however, the selective antagonist APV had no effect on fast Ca^2+^ transients in MG neurons evoked by 20× or 1× stimulation (Swaminathan et al., 2019). These findings indicate that the evoked Ca^2+^ responses and the spontaneous Ca^2+^ transients observed in cholinergic MG neurons depend on similar synaptic transmission mechanisms as seen by a requirement for activation of presynaptic Ca^2+^ channels and by a shared dependence on postsynaptic nAChRs or/and 5-HT_3_Rs, but likely without involvement of GABA_A_, NMDA, or AMPA receptors. Whether purinergic (P2X) receptors contribute to evoked synaptic responses in mouse colon remains an open question. In guinea pig ileum 67% of S-type MG motor and interneurons (most of which are cholinergic), display fast EPSPs mediated by nAChRs and P2X receptors, but such responses are much less abundant in the colon (Galligan et al., 2000) and were not investigated here.

Calculating the delay times of Ca^2+^ responses evoked by 1× stimulation provided additional insights concerning the synaptic pathways leading to MG neuron activation (Figure 5). The first step involved measuring the time elapsed between stimulus application and response onset, defined as a two to fourfold increase in basal ΔF/F_0_. By next subtracting the time required for impulse propagation to the MG estimates of synaptic delay were obtained. Such estimates depend on knowing the conduction velocity of axons projecting longitudinally from the stimulation site to the MG. Our reliance on a previously published conduction velocity value of 0.55 mm/ms is reasonable because it is based on longitudinally projecting MP axons and because the authors used intracellular methods to conduct their measurements (Stebbing and Bornstein, 1996). While synaptic delay times were broadly distributed from 1.9 to 65 ms, the largest fraction were <4 ms, values consistent with monosynaptic transmission (Katz and Miledi, 1965; Goyal and Chaudhury, 2013) and indicative of direct inputs to MG neurons from nearby ganglia.

In addition to its utility in assessing the properties of synapses on cholinergic MG neurons, GCaMP6f Ca^2+^ imaging can identify regulatory influences on such synapses. α7-nAChR and PACAP agonists were chosen as potential regulators because of their demonstrated actions at synapses in autonomic ganglia and brain (McGehee et al., 1995; Gray et al., 1996; McGehee and Role, 1996; Pugh et al., 2009; Jayakar et al., 2014). In addition, α7-nAChR subunit protein (Obaid et al., 2005) and transcripts (Supplementary Figure 2C) are detected in MG, and PACAP and PAC_1_Rs are present in MP neurons and fibers (Portbury et al., 1995; Miampamba et al., 2002). The acute modulation of F_S_ by GTS-21 (Figures 6A–D) required α7-nAChR activation because GTS-21 is an α7-nAChR selective agonist (Briggs et al., 1997; Meyer et al., 1997; Nai et al., 2003) and because the GTS-21 induced increase in F_S_ was blocked by the α7-nAChR selective antagonist MLA. GTS-21 appeared to act on presynaptic terminals rather than on somatic α7-nAChRs because α7-nAChR antagonists (MLA and α-bungarotoxin) previously failed to block ACh induced responses in MG neuron somas (Zhou et al., 2002) and in our studies GTS-21 failed to evoke appreciable somatic Ca^2+^ signals while inducing robust increases in F_S_. Moreover, α7-nAChRs display an immunolabeling pattern consistent with expression on presynaptic terminals in MG (Obaid et al., 2005). The increase in F_S_ due to presynaptic α7-nAChR activation is consistent with enhanced neurotransmitter release demonstrated in brain and sympathetic ganglia (McGehee et al., 1995; Gray et al., 1996; McGehee and Role, 1996) and suggested for enteric neurons (Obaid et al., 2005) and may serve to increase the reliability of synaptic transmission (Chang and Berg, 1999). Contrasting with the short-lived actions of GTS-21, PACAP induced increases in Ca^2+^ transient F_S_ remained evident for at least 60 min. Such persistence is consistent with a GPCR mediated signaling pathway that activates effectors having extended actions on downstream synaptic targets. Studies with parasympathetic neurons indicate that PACAP acting *via* PAC_1_Rs triggers a cascade of intracellular changes involving cyclic AMP/protein kinase A and neuronal nitric oxide synthase effectors, thereby enhancing quantal ACh release from presynaptic terminals to increase the frequency of spontaneous synaptic currents by >200% and increase their amplitudes by ≈40% (Pugh et al., 2009; Jayakar et al., 2014). The PACAP-induced increase in Ca^2+^ transient F_S_ seen here is consistent with an effect on presynaptic processes that enhance transmitter release at MG synapses. In the amygdala, PACAP causes rapid, persistent enhancement of excitatory transmission to neurons in the central lateral nucleus *via* protein kinase A dependent signaling, but does so by a postsynaptic mechanism featuring a ≈30% increase in evoked AMPAR mediated EPSC amplitude (Cho et al., 2012). It remains possible that PACAP induces a comparable 30–40% increase in MG neuron synaptic responses that is not reflected in a detectable increase in Ca^2+^ transient A_S,Peak_.

## Conclusion

In summary, our results reveal that targeted GCaMP6f Ca^2+^ imaging can be used to assess the components, function and modulation of synapses on cholinergic MG neurons. The advantage of GCaMP6 Ca^2+^ imaging is its utility in assessing intact neural circuits such as the colonic MP, an approach that reveals abundant and diverse synaptic interactions. While based on myenteric neurons, the results further indicate that GECIs can be used to probe the function and modulation of synapses targeted at specific classes of neurons throughout the nervous system.

## Data Availability Statement

The original contributions presented in the study are included in the article/Supplementary Material, further inquiries can be directed to the corresponding author/s.

## Ethics Statement

The animal study was reviewed and approved by the University of Toledo Institutional Animal Care and Use Committee (IACUC).

## Author Contributions

JM directed the project, conducted the imaging experiments using GCaMP6f expressed in ChAT+ mice, analyzed the results, wrote the manuscript, and corresponded with the journal staff. KS-E conducted the imaging experiments using GCaMP6s expressed in E2a mice and analyzed the results. AN-K conducted the confocal imaging studies to document the presence of nAChR subunits and serotonin. BD provided intellectual input and advised KS-E. KA conducted RNA-scope experiments. MH secured NIH funding and conducted RNA-scope and immunolabeling experiments. All authors contributed to the article and approved the submitted version.

## Conflict of Interest

The authors declare that the research was conducted in the absence of any commercial or financial relationships that could be construed as a potential conflict of interest.

## Publisher’s Note

All claims expressed in this article are solely those of the authors and do not necessarily represent those of their affiliated organizations, or those of the publisher, the editors and the reviewers. Any product that may be evaluated in this article, or claim that may be made by its manufacturer, is not guaranteed or endorsed by the publisher.

## Footnotes

1
https://www.janelia.org/jgcamp8-calcium-indicators#cultured_neuron
