# Supplementary material for: Synaptic Components, Function and Modulation Characterized by GCaMP6f Ca2+ Imaging in Mouse Cholinergic Myenteric Ganglion Neurons
Source: Front Physiol. 2021 Aug 2;12:652714. doi: 10.3389/fphys.2021.652714 (PMC8365335; doi:10.3389/fphys.2021.652714)
Supplement: Supplementary file 1 [file Data_Sheet_1.zip › Presentation 2/Figure S2 Caption.DOCX]

**Supplementary Figure 2.** Expression of neurotransmitter protein and receptor mRNA transcript in mouse colon MG. **A.** Punctate 5-HT immunoreactivity (Red; labeled with anti-5HT antibody) is evident adjacent to MG neuron somas (Blue; labeled with anti-neuron specific HuC/D antibody). **B** and **C**. Subunit specific RNAscope probes detected punctate expression of mRNA transcripts encoding α3- and α7-nAChR subunits in MG neurons (Red in **B** and **C**, respectively). DAPI (Blue) labeling identifies cell nuclei. For all three markers, labeling was dense in the myenteric plexus with little obvious labeling in the connective tissue or epithelium. Scale bars in A-C represent 40 μm. **D.** Hematoxylin and eosin section of colon shows the location of the MG between the inner circular (ICM) and outer longitudinal muscle (OLM) layers of the colon wall and the underlying epithelium (Epith).
