# Supplementary material for: Synaptic Components, Function and Modulation Characterized by GCaMP6f Ca2+ Imaging in Mouse Cholinergic Myenteric Ganglion Neurons
Source: Front Physiol. 2021 Aug 2;12:652714. doi: 10.3389/fphys.2021.652714 (PMC8365335; doi:10.3389/fphys.2021.652714)
Supplement: Supplementary file 1 [file Data_Sheet_1.zip › Presentation 2/Figure S3 Caption.DOCX]

**Supplementary Figure 3.** Functional GABA_A_Rs, evident on some cholinergic MG neurons, do not contribute to spontaneous or evoked synaptic events. **A.** Time course of GCaMP6f mediated Ca^2+^ fluorescence changes in 10 neuron somas (Black traces) in response to focally applied GABA (Black bar, 100 µM). Red triangles above the traces depict the timing of peak responses (A_A,Peak_). The response average from these neurons and of those after incubation with bicuculline (10 μM) are depicted by the blue and purple traces, respectively. **B.** GABA response parameters. Overall 18 ± 2% of cholinergic MG neurons (n = 338, N = 22) responded to GABA application. *Left:* Average A_A,Peak_ responses to 100 µM GABA (0.121 ± 0.033, n = 30; N = 2) or 1 µM GABA (0.095 ± 0.012, n = 308, N = 20) were indistinguishable (*p > 0.05*). *Right:* Incubation with the GABA_A_R selective antagonist Bicuculline (20 µM; 10-15 min) reduced the A_E,Peak_ response of cholinergic MG neurons (n = 132; N =9) to 1 µM GABA by 47 ± 7% relative to the same neurons tested before antagonist application (*p < 0.05*; Black bar, Control). **C.** GABA_A_Rs do not contribute to the synapse-dependent spontaneous Ca^2+^ transients. Graphs depict spontaneous Ca^2+^ transient frequency (F_S_, Left panel) and amplitude (A_S,Peak_, Right panel) measurements from n = 30 cholinergic neuron pairs in N = 2 ChAT^+^/ GCaMP6f^+^ MG explants tested before (Control, Gray bars) and after treatment with 10 µM Bicuculline (Purple bars). Bars represent group F_S_ and A_S,Peak_ averages while the superimposed dot plots and connecting lines depict their values from individual neurons before and after treatment. Neither F_S_ nor A_S,Peak_ were detectably changed by inhibiting GABA_A_Rs (*p > 0.05* for both). **D.** GABA_A_Rs do not contribute to the stimulus-evoked synaptic Ca^2+^ responses. ΔF/F_0_ responses (A_E.Peak_) evoked by 20X MG connective stimulation cholinergic MG neurons (n = 30, N = 2) assayed before (Control, Black bar) and after treatment with 10 µM Bicuculline (Purple bar) were not detectably different (*p > 0.05*).
