# Supplementary material for: Synaptic Components, Function and Modulation Characterized by GCaMP6f Ca2+ Imaging in Mouse Cholinergic Myenteric Ganglion Neurons
Source: Front Physiol. 2021 Aug 2;12:652714. doi: 10.3389/fphys.2021.652714 (PMC8365335; doi:10.3389/fphys.2021.652714)
Supplement: Supplementary file 1 [file Data_Sheet_1.zip › Presentation 2/Video 1.DOCX]

**Supplementary Figure 1.** The time-lapse video depicts spontaneous changes in GCaMP6f Ca^2+^ fluorescence (Ca^2+^ transients) in neurons within two MG. ROIs drawn around 15 neuron somas chosen for analysis are indicated in red. Neurons labeled 1, 2, and 3 displayed isolated (1) moderately clustered (2) or extremely clustered (3) Ca^2+^ transient activity as depicted in Fig. 2B.
