# Supplementary figures and images for: Synaptic Components, Function and Modulation Characterized by GCaMP6f Ca2+ Imaging in Mouse Cholinergic Myenteric Ganglion Neurons
Source: Front Physiol. 2021 Aug 2;12:652714. doi: 10.3389/fphys.2021.652714 (PMC8365335; doi:10.3389/fphys.2021.652714)

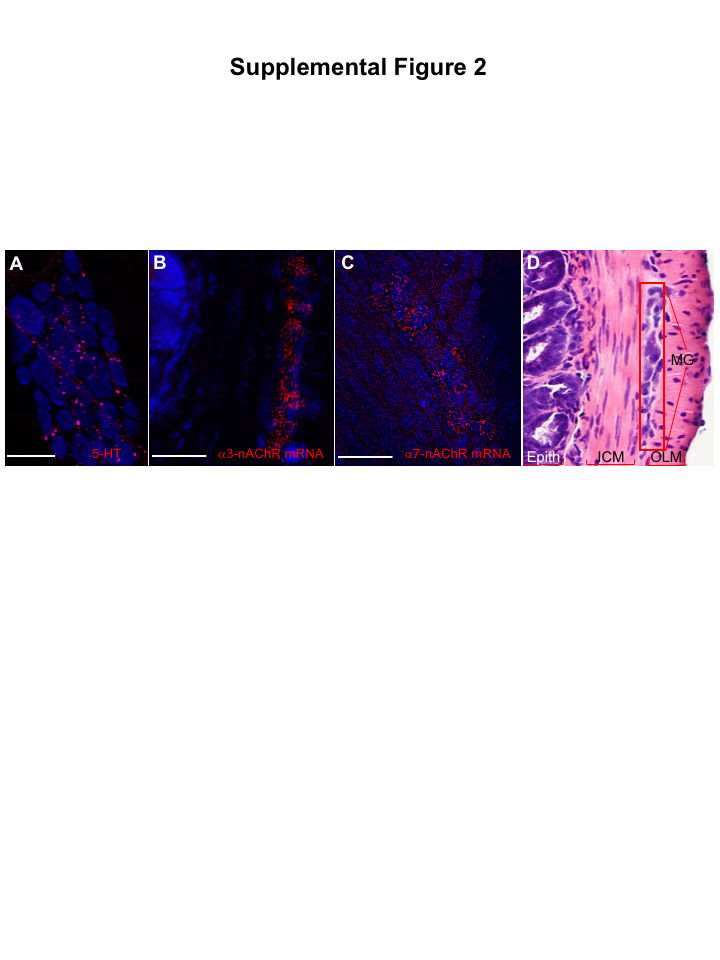

Supplement: Supplementary file 1 [file Data_Sheet_1.zip › Presentation 2/Figure S2.TIFF]

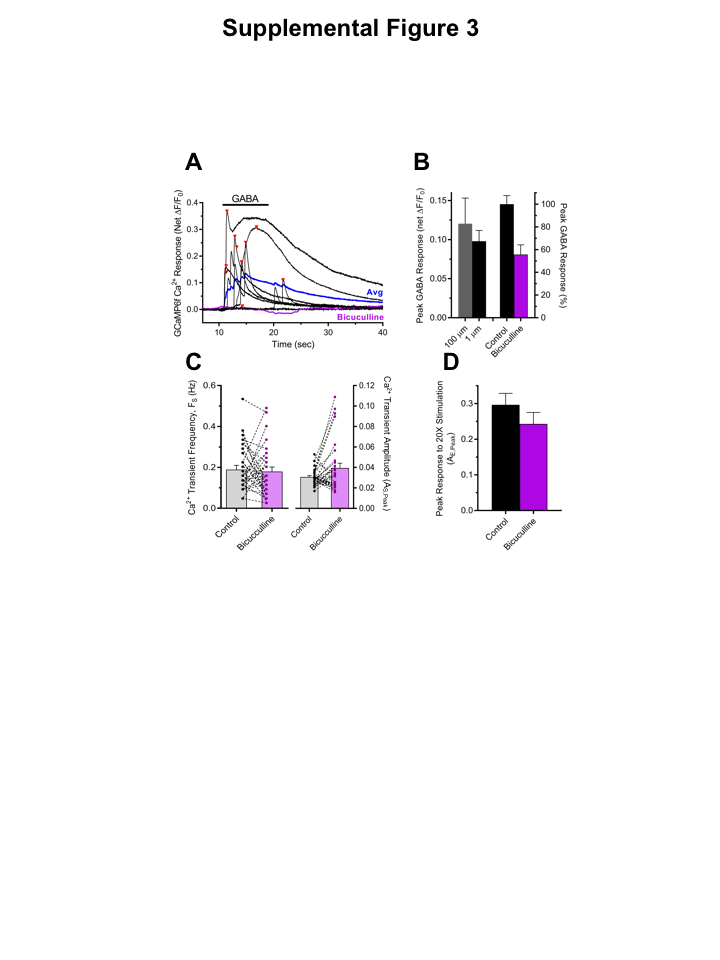

Supplement: Supplementary file 1 [file Data_Sheet_1.zip › Presentation 2/Figure S3.TIFF]
